# A Systematic Review of Individual and Contextual Factors Affecting ART Initiation, Adherence, and Retention for HIV-Infected Pregnant and Postpartum Women

**DOI:** 10.1371/journal.pone.0111421

**Published:** 2014-11-05

**Authors:** Ian Hodgson, Mary L. Plummer, Sarah N. Konopka, Christopher J. Colvin, Edna Jonas, Jennifer Albertini, Anouk Amzel, Karen P. Fogg

**Affiliations:** 1 Independent Consultant, Bingley, United Kingdom; 2 Independent Consultant, Dar es Salaam, Tanzania; 3 Center for Health Services, Management Sciences for Health, Arlington, Virginia, USA; 4 Centre for Infectious Disease Epidemiology and Research (CIDER), Division of Social and Behavioural Sciences, School of Public Health and Family Medicine, University of Cape Town, Cape Town, South Africa; 5 United States Agency for International Development (USAID)/Africa Bureau, Washington, D.C., USA; 6 USAID/Bureau for Global Health (BGH)/Office of HIV/AIDS, Washington, D.C., USA; 7 USAID/BGH/Office of Health, Infectious Diseases, and Nutrition, Washington, D.C., USA; University of Southampton, United Kingdom

## Abstract

**Background:**

Despite progress reducing maternal mortality, HIV-related maternal deaths remain high, accounting, for example, for up to 24 percent of all pregnancy-related deaths in sub-Saharan Africa. Antiretroviral therapy (ART) is effective in improving outcomes among HIV-infected pregnant and postpartum women, yet rates of initiation, adherence, and retention remain low. This systematic literature review synthesized evidence about individual and contextual factors affecting ART use among HIV-infected pregnant and postpartum women.

**Methods:**

Searches were conducted for studies addressing the population (HIV-infected pregnant and postpartum women), intervention (ART), and outcomes of interest (initiation, adherence, and retention). Quantitative and qualitative studies published in English since January 2008 were included. Individual and contextual enablers and barriers to ART use were extracted and organized thematically within a framework of individual, interpersonal, community, and structural categories.

**Results:**

Thirty-four studies were included in the review. Individual-level factors included both those within and outside a woman’s awareness and control (e.g., commitment to child’s health or age). Individual-level barriers included poor understanding of HIV, ART, and prevention of mother-to-child transmission, and difficulty managing practical demands of ART. At an interpersonal level, disclosure to a spouse and spousal involvement in treatment were associated with improved initiation, adherence, and retention. Fear of negative consequences was a barrier to disclosure. At a community level, stigma was a major barrier. Key structural barriers and enablers were related to health system use and engagement, including access to services and health worker attitudes.

**Conclusions:**

To be successful, programs seeking to expand access to and continued use of ART by integrating maternal health and HIV services must identify and address the relevant barriers and enablers in their own context that are described in this review. Further research on this population, including those who drop out of or never access health services, is needed to inform effective implementation.

## Background

HIV is responsible for a large proportion of indirect maternal deaths in countries with high HIV prevalence [1]–[3]. New [1] analyses reveal that there is wide range in the estimated impact of HIV on pregnancy-related and maternal mortality from 24 percent of pregnancy-related deaths [4] to 6.4 percent of maternal deaths [5] in sub-Saharan Africa. Globally, estimates range from roughly 21 percent to 0.4 percent of maternal deaths [6] that are related to HIV. However, the impact of HIV on pregnancy and maternal mortality is substantial for countries with high HIV prevalence [7]. Hospital-based studies in Africa have shown relative risks of pregnancy-related death among HIV-infected women ranging from two to eight-times greater than in non-infected women [8]. There is also evidence of increased risk of direct obstetric complications, such as sepsis, among HIV-infected pregnant women [8], [9].

In countries where HIV is highly prevalent, HIV infection is a leading cause of pregnancy-related deaths and has even reversed gains in reducing maternal mortality [2]. Many of these countries struggle to maintain adequate health system capacity to meet the associated service needs. Antiretroviral therapy (ART) is effective in reducing maternal mortality among HIV-infected women [10], but ART initiation, adherence, and retention in care remain problematically low in some regions, even when ART is available [11]. There is an urgent need to understand the factors affecting the uptake of this critical intervention in order to improve programming and extend the reach of services and supportive interventions to this population.

This review is one of three systematic reviews that together consider evidence around efforts to reduce mortality among HIV-infected pregnant and postpartum women. One review assesses the evidence on the effectiveness of interventions to decrease death and morbidity among HIV-infected women during pregnancy and up to one year postpartum [12]; another review examines the health system barriers and enablers to ART initiation, adherence, and retention and evidence on health system interventions that may facilitate access to maternal ART [13]. This current review synthesizes evidence on the individual and contextual barriers to and enablers of ART initiation, adherence, and retention among the same population. This systematic review was guided by the following question:

What are the individual and contextual factors affecting the initiation, adherence, and retention to ART among HIV-infected pregnant women during and following pregnancy?

Factors of interest include those individual, interpersonal, community, and structural forces which influence an HIV-infected woman's ability to initiate, adhere to, or be retained in ART care. Some health system factors identified in the other above-mentioned review are closely related to contextual factors identified in this review. We have included information on health systems factors if they capture the woman’s perspectives on or experiences of health system issues.

## Methodology

### Review Design

We undertook a systematic review of both qualitative and quantitative evidence of individual and contextual factors that inhibit or enable access to and use of ART for HIV-infected pregnant and postpartum women. Study findings were analyzed thematically and we used a conceptual framework for data extraction and synthesis that was informed by the World Health Organization (WHO) health systems framework [14] and Supporting the Use of Research Evidence (SURE) framework [15].

### Study Eligibility

#### Inclusion Criteria

To maximize the breadth of the study findings, we included any study that reported empirical qualitative or quantitative findings relevant to the review question. Studies from low- and middle-income countries (LMICs), as well as high-income countries, were included, as were studies conducted in community or health system settings. Due to time and resource constraints, we only included studies written in English. To maximize the relevance of the study findings to current maternal ART policy and practice, we only included studies published between January 1, 2008 and March 26, 2013.

Because this review was conducted in parallel with a separate but linked systematic review of health systems factors affecting ART initiation, adherence, and retention for pregnant and postpartum women [13], we only included studies that described health systems-related factors if these were described from the woman’s perspective or experience. For example, long waiting times at health facilities are a contextual barrier from women’s perspectives when they do not attend services because they do not believe they have enough time to wait. Long waiting times may also reflect broader supply-side issues within the health system, such as inefficient models of care or system-level resource constraints.

#### Exclusion Criteria

Studies were excluded if they focused on HIV-infected pregnant or postpartum women on ART and/or in PMTCT programs but did not identify individual or contextual barriers or enablers of ART initiation, adherence, or retention. We excluded studies that reported on relevant health systems barriers and enablers if there was no discussion of these factors from the perspective of pregnant women. We also excluded studies of broader cohorts of people with HIV (e.g., all adults on ART) if barriers and enablers specific to pregnant or postpartum women could not be distinguished in the findings.

### Search Strategy and Selection Process

#### Search strategy

Both peer-reviewed journal articles and gray literature were searched to identify eligible studies. Peer-reviewed journal articles were searched systematically in the PubMed and Social Sciences Citation Index (SSCI) databases using variations of three key terms:

• Population of interest (i.e., pregnant women and postpartum women infected with HIV);
• Intervention of interest (i.e., ART);
• Outcomes of interest (i.e., initiation, adherence, retention).

A full search strategy for one of the database searches can be found in the Supporting Information. Gray literature was also searched on relevant conference abstract databases, multilateral and bilateral agency websites, and websites of non-governmental organizations (NGOs) conducting research or implementing programs of relevance. Articles and abstracts were excluded if they did not address the population, interventions or outcomes of interest.

#### Study selection

Studies were selected for review in two stages. First, three review authors independently assessed the first 100 abstracts retrieved from PubMed. Each reviewer’s list of selected articles and accompanying rationale was compared with the list from the other reviewers; discrepancies were discussed and resolved. Inclusion and exclusion criteria were refined and clarified during this process. In the second step, one review author (SK) reviewed the remaining abstracts and included or excluded studies.

### Quality Assessment and Data Extraction

#### Characterizing the evidence base

In order to assess the strength of the underlying evidence base for the review, we first developed an overview of key characteristics of the included studies by summarizing several variables, including study design, sample size and strategy, geographic region, healthcare setting, and risk of bias. We then ranked each included study as low, medium, or high with respect to overall risk of bias. Given the diversity of study designs included and the difficulty of comparing study quality assessments across widely varying study types, these rankings were based on criteria appropriate to each study design (e.g. different quality appraisal criteria were used for qualitative and quantitative studies). These rankings were further justified via short narratives. This provided an overview of the quality of the existing evidence base, as represented by the included studies. No studies were excluded on the basis of the quality assessment. Rather the quality assessment process was used to identify weaknesses in study methodologies and to guide the interpretation and assessment of study findings.

#### Data extraction and management

Once the study selection process was concluded, one review author (SK) extracted data from the studies using a standard template. Initial data extraction captured both the study characteristics (e.g., setting, participants, and type of ART program reviewed) as well as key findings related to factors associated with initiation, adherence, and retention of ART. A second author (IH) also reviewed the studies and extracted data relating to key individual and contextual barriers and enablers associated with initiation, adherence, and retention. Extracted findings from both authors were compared and discrepancies resolved.

### Data Synthesis

The barriers and enablers identified were arranged thematically within a framework of individual, interpersonal, community, and structural categories [16]. These categories were further divided into enabling factors and barriers to ART adherence, e.g., knowledge about HIV or ART or wanting to protect one’s child (individual-level enablers); domestic violence or spousal dependence (interpersonal-level barriers); stigma (community-level barrier); or health worker attitude or support group participation (structural-level enablers). This framework was reviewed by all review authors for accuracy and comprehensiveness.

The intention of the analysis process was to produce a detailed list of factors that have been reported as affecting the three ART outcomes of interest for pregnant and postpartum women, and to offer, when possible, brief explanations for how these factors might operate and in which contexts they were most salient. Given the diversity of disciplinary approaches, sociocultural contexts, study questions, and study designs in this review’s included studies and the broad scope of the review question, it was not possible to develop the analysis of these factors further than a straightforward thematic analysis. The intention, therefore, is not to provide a rich explanatory model for how each of these factors might work, or to develop a global theory of barriers and enablers to maternal ART. Rather, the analysis is intended to provide policymakers and practitioners with a roadmap for how to think about, and where to look for, the factors that might shape pregnant and postpartum women’s access to ART.

As a final step in the analysis process, we assessed our confidence in each of the “key review findings” by describing the strength and generalizability/transferability of the evidence supporting that finding. For each review finding, therefore, we looked back at the studies that contributed to that finding and considered: 1) how strong the underlying study design was, 2) what the risk of bias was, 3) what level of detail and/or context was provided to enable interpretation, and 4) how frequently the review finding was found across the individual studies. This approach is modeled on the CERQual approach to assessing the confidence in findings of qualitative systematic reviews [17]. We then ranked the strength of the evidence underlying each finding as high, moderate or low.

We were also interested in the generalizability or transferability–depending on whether we were considering quantitative or qualitative evidence–of the evidence with respect to public sector health services in low and middle-income countries with high HIV prevalence (where most of the global burden lies and where women and programs need the most support). The concept of ‘transferability’ is used in assessing qualitative research findings as a more appropriate alternative to the concept of ‘generalizability’ [18]. Transferability expresses the degree to which the study authors have provided contextual information and other forms of ‘thick description’ that allow the reader to determine to which other contexts particular findings might be transferable. For each key review finding, we therefore asked two additional questions: 5) how many of the studies supporting this finding were conducted within existing services settings, and 6) how many came from LMICs with high HIV prevalence. Here too, we ranked generalizability or transferability as high, moderate or low.

Findings on the strength and generalizability or transferability of review findings to high prevalence settings have been provided below along with a brief narrative justification for each. No studies were excluded based on the quality appraisals of the studies, but most studies used to illustrate core findings in the text were supported by strong evidence.

## Results

### Overview of Studies Included and ART Regimens

**Figure 1:**
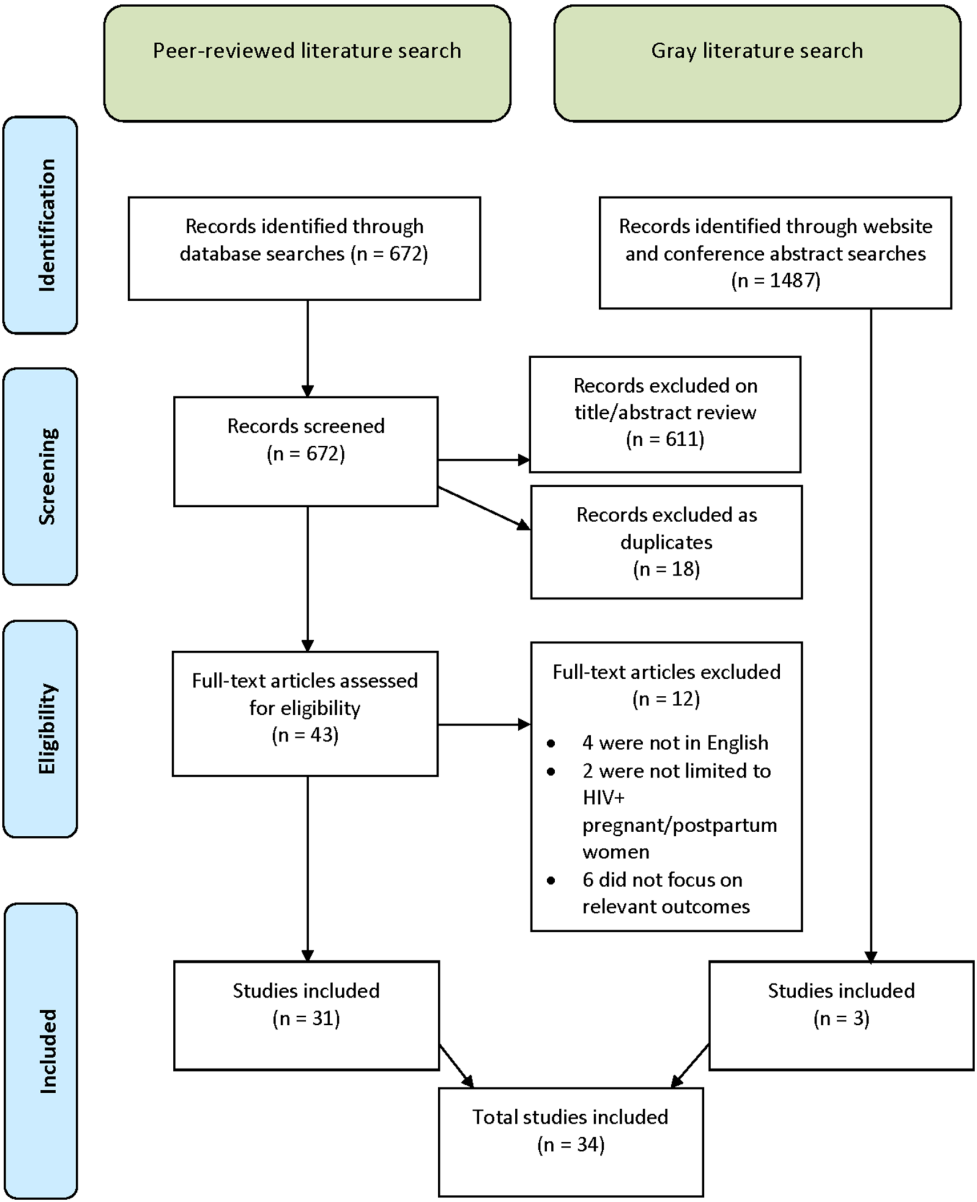
Flow Diagram for Study Search and Inclusion.

The results of the peer-reviewed journal article and gray literature search are summarized in the flow diagram in Figure 1. The peer-reviewed journal article search yielded 672 articles, of which 31 were included in the core review. A total of 1,487 grey literature documents were assessed, of which three were selected for inclusion in the review.

Thirty-four studies met the inclusion criteria for this review [19]–[52]. Table 1 provides a brief overview of the key characteristics of the included studies. A more detailed table is available in the Supporting Information that provides information on each of the studies reviewed, including each study’s location, design, population size, type of ART program, and key findings related to initiation, adherence and retention during pregnancy and postpartum.

**Table 1:** Summary of Key Characteristics of Included Studies.

Characteristics		Number of studies
**Regions**		
	Sub-Saharan Africa	27
	Asia	1
	Latin America	2
	Europe/North America	4
	Middle East	0
**Geographic Setting**		
	Rural	6
	Urban	12
	Both	11
	Unclear	5
**HIV Prevalence Rates**		
	Low (0–5%)	9
	Moderate (5–15%)	11
	High (15% or higher)	14
**Study Designs**		
	Quantitative methods	16
	Qualitative methods	12
	Mixed methods	6
**Explicit Intervention Tested**		
	Yes	4
	No	30

Twenty-seven of the studies were carried out in sub-Saharan Africa, one in Asia, two in Latin America, and four in Europe and the US. Sixteen studies used quantitative methods, twelve used qualitative methods and six employed both qualitative and quantitative methods. Most study participants were HIV-infected pregnant or postpartum women. Eight studies included data from interviews or focus group discussions with health care workers, community members, partners, and/or family members of HIV-infected pregnant and postpartum women.

Four studies described and evaluated interventions. These include: a study from Zambia exploring the benefits of couple counseling for initiation [32]; a study from South Africa describing the impact of text messaging on promoting positive health choices [52]; another study from South Africa describing the benefits of rapid ART initiation among pregnant women [44]; and a study from Malawi exploring the use of community health workers in promoting ART adherence [34].

There were a number of different prevention of mother-to-child transmission (PMTCT) and maternal ART regimens used in the included studies. Seven studies involved women who had initiated ART for therapeutic reasons before they enrolled in a PMTCT program. Twenty-one studies included participants not on ART who then enrolled in PMTCT programs, and examined factors influencing ART initiation, adherence and/or retention during pregnancy and, depending on regimen, for a brief period thereafter. The regimens used in these studies included use of single-dose nevirapine (sdNVP), Option A, and Option B regimens (described in more detail in Table 2) [53]–[55].

**Table 2:** Antiretroviral medication regimens in included studies.

Regimen	Purpose: PMTCT Prophylaxis*	Purpose: Treatment for the Mother	Notes
**Single-dose** **nevirapine** **(sdNVP)**	One intrapartum dose taken at thebeginning of a woman’s labor	N/A	Introduced in 2000, this regimen is no longer recommended by WHO unless as part of combination PMTCT (Option A)
**Option A**	**For pregnant women living** **with HIV with CD4>350**	**For pregnant women with** **CD4 cells <350 or clinical** **Stage 3–4 disease**	
	(a) Antepartum: Antenatal zidovudine(AZT) twice daily starting as early as14 weeks gestation	Triple antiretroviral medications (ARVs) often combined within a single pill (a “fixed dose combination”) that is taken twice daily, starting as soon asdiagnosed and continued for life	
	(b) Intrapartum: at onsent of labor, sdNVP and AZT every 3 hours and lamivudine (3TC) every 12 hours until delivery		
	(c) Postpartum: twice daily AZT/3TC for 7 days		
**Option B**	Triple ARVs starting as early as 14 weeks gestation and continuedintrapartum and throughchildbirth if not breastfeedingor until 1 week after cessation of breastfeeding	Triple ARVs starting assoon as diagnosed,continued for life	Under WHO’s 2010 PMTCT ARV guidance, countries have the option to choose between two prophylaxis regimens for pregnant women living with HIV: Option A and Option B.
**Option B+**	Triple ARVs starting as soon asdiagnosed, continued for life	Triple ARVs starting assoon as diagnosed,continued for life	Option B+ was conceived and implemented in Malawi in 2011. In April 2012, WHO released a programmatic update in which it urged countries to consider Option B and B+

*PMTCT prophylaxis refers to the use of ARV drugs solely for the purpose of reducing the risk of vertical transmission when a woman is not on standard ART for therapeutic reasons.

Six studies involved women who were initiated on ART for therapeutic reasons after being enrolled in a PMTCT program. These women were eligible to initiate ART because of their advanced clinical disease and/or high CD4 count. All of the reviewed studies preceded the introduction of Option B+ (where all pregnant HIV-infected women are initiated/supported on lifelong ART regardless of WHO clinical stage and/or CD4 count). Thus, no reviewed studies examined ART initiation, adherence, or retention within an Option B+ program.

The one-time nature of the sdNVP-based PMTCT regimen means that it has only limited comparability to situations in which a woman might begin long-term or life-long ART during pregnancy. Papers reporting on sdNVP-based programs were excluded from the review unless they addressed barriers and enablers relevant to initiation, adherence or retention from the woman’s point of view; seven such sdNVP-focused studies were included.

### Overview of Barriers and Enablers Identified

Individual and contextual factors influencing ART initiation, adherence, and retention for pregnant and postpartum women were identified through qualitative participant reports, study author observations and interpretations, and quantitative measures of association reported in the studies. Data were extracted from both the findings sections of study reports as well as ‘second-order’ author interpretations of findings [56] in the discussion sections of papers.

Key barriers and enablers to ART initiation, adherence, and retention identified in the studies were categorized thematically within individual, interpersonal, community, and structural levels of influence. This approach to organising and understanding the factors shaping health practices, processes and outcomes is typically defined as the ‘socio-ecological’ approach [57]–[59]. While the findings have been divided into these four different levels of analysis, they are of course not all independent, and in fact many factors identified at one level may interact with other factors within and between levels (e.g., fear of stigma at the individual level and norms around non-disclosure at the community level).

An overview of the key identified factors is presented in Table 3. This table summarizes enablers and barriers separately, by the relevant outcome of interest. A more comprehensive table is available in the Supporting Information that provides the same information in more detail, showing the hierarchy of main themes and subcategories, and, for each finding, which studies contributed to the finding. The assessment of our confidence in these findings is provided in Table 4.

**Table 3:** Summary of ART Enabler and Barrier Findings, by Level and Outcome of Interest.

	Initiation		Adherence		Retention	
	Enabler	Barrier	Enabler	Barrier	Enabler	Barrier
**Individual**	• Knowledge of PMTCT and referral process (higher)	• Age (lower)	• Age (higher)	• Age (lower)	• Sufficient knowledge of PMTCT	• Poor knowledge of ART
•	• Desire to protect child	• Knowledge of PMTCT (lower)	• Education level (higher)	• Education level (lower)	• Access to cell phone (text reminders/appointments)	• HIV denial
•	• Education level (higher)	• Denial of HIV	• Sufficient knowledge of PMTCT	• Rural residency	• Religion	• Scheduling problems
		• Fear of job loss	• Desire to remain healthy	• HIV denial		• Religion
		• Reluctance to start lifelong treatment	• Desire to protect child	• Concern ART will harm child		
		• Forgetting medication		• Conflict with role as homemaker		
		• Scheduling problems		• Misplacing medication		
		• Feeling too healthy		• Forgetting medication		
				• Away from home		
				• Lack of food/water/income		
				• Religion		
				• Use of drugs/alcohol		
**Interpersonal**	• Partner involved in care	• Dependence on or permission needed from partner	• Disclosure to partner	• Dependence on or permission needed from partner	• Disclosure to partner	• Dependence on or permission needed from partner
		• Non-disclosure to partner	• Partner involved in care	• Fear of domestic violence after disclosure	• Partner not involved in care	• Fear of domestic violence after disclosure
		• Partner not involved in care	• Family support	• No family support		
		• No support from family		• Relatives ‘stealing’ ART pills		
**Community**		• Actual or anticipated stigma	• Disclosure without stigma	• Actual or anticipated stigma		• Actual or anticipated stigma
**Structural**	• Support group participation	• Low attendance at ANC	• Receiving other treatment (e.g., for tuberculosis) or vitamin supplements	• Actual or anticipated breach of confidentiality in health center	• First pregnancy registration	• Late disengagement (within 30 days of delivery)
	• Treatment support or counseling	• Negative health worker attitudes	• Enrolment in ART pre-delivery	• Payment problems	• Community health worker involvement	• Low or late attendance at ANC
	• Encouragement from a traditional birth attendant	• Long queues at health center		• Negative health worker attitudes	• Successful completion of PMTCT pre-delivery	• Negative health worker attitudes
		• Transportation problems		• Long queues at health center	• Enrolment in ART pre-delivery	• Actual or anticipated breach of confidentiality
				• Medications not dispensed correctly		• Long queues at health center
				• Transportation problems		• Transportation problems

**Table 4:** Strength of Evidence and Generalizability/Transferability of Key Review Findings to High Prevalence Contexts.

Level of Influence	Key Review Finding	Strength of Evidence Summary	Generalizability/Transferability Summary
**Individual**	1a) Socio-demographic factors (i.e., age, educational level, residency) can influence ART initiation, adherence, and retention.	**High** Eight papers reported a range of socio-demographic factors. Age and educational level were most widely reported as specific findings in papers with strong quantitative designs, specifically that older women, or those achieving a higher level of education, were more likely to adhere. Residency was reported in two papers – one quantitative (women in central hospitals were less likely to disengage than women in smaller and more remote hospitals), and one qualitative/descriptive, focusing on challenges in rural communities around maternal roles.	**Moderate** This finding was reported across a range of contexts, and is generalizable, although issues around maternal role may be context-based.
	1b) Level of knowledge about health services, ART, and/or PMTCT can affect ART initiation, adherence, and retention.	**Moderate** Six papers reported on the association between knowledge (of health services, or ART) and adherence. Mixed method and quantitative designs reported higher levels of knowledge correlating with adherence. One qualitative study based on focus group discussions and one paper derived from a conference abstract confirmed the other studies’ findings. Knowledge of the referral process was only cited as an enabler in one mixed method paper.	**High** This finding was reported across a range of contexts, with no anomalies, and is likely to apply broadly, although evidence for knowledge of the referral process requires further exploration.
	1c) Women’s fears and perceptions of treatment, and the desire to maintain their roles and status within families, can affect ART initiation, adherence, and retention.	**Moderate** Eleven papers reported on these issues. Qualitative and mixed method studies with strong designs reported that a woman’s role in the family may conflict with her needs as a patient. A desire to protect children from HIV was reported in qualitative studies and one quantitative study, although the latter was only a second order interpretation.	**Moderate** Evidence relating to these factors was internally consistent within the review, although it did include second order interpretations. The particular forms these factors take are likely to be context-based.
	1d) Factors in a woman’s daily life can affect ART initiation, adherence, and retention.	**High** Challenges managing the practical demands of ART were reported in 12 quantitative or mixed method papers, and included day-to-day demands or (in one mixed method paper) lack of access to water and/or food. Scheduling problems, or being away from home, were frequently reported barriers to adherence. All findings were of first order interpretation.	**Moderate** Evidence relating to these factors was internally consistent within the review, although the particular forms these factors take are likely to be context-based.
	1e) Beliefs (e.g., religious beliefs, feeling healthy, and having a positive outlook) can affect ART initiation, adherence, and retention.	**Moderate** Seven papers reported that individual beliefs affected initiation, adherence, and retention. The finding about feeling too well to attend HIV services was only a second order interpretation in one quantitative paper and a report by one respondent in a qualitative study. Feeling ‘happy’ was significant in one quantitative study. The positive role of religion was a strong finding in two papers, though one (quantitative) was a second order interpretation. Advice to use traditional medicines instead of ART was only cited as a barrier in only one mixed method study, but it was a core finding.	**Moderate** Evidence was reported across a range of contexts, confirming the relevance of the broad finding, but specific examples are likely to be context-dependent.
	1f) Behavioral factors can be key barriers to ART initiation, adherence, and retention.	**High** Behavioral factors influencing ART initiation, adherence, and retention were reported in eight papers. Three quantitative papers with strong designs reported use of alcohol or illegal drugs as barriers to adherence. Four quantitative studies, and one mixed method study, reported evidence that forgetting, or misplacing, medication was a barrier to adherence.	**High** This finding was reported across a wide range of contexts; the two essential components can be generalized.
**Interpersonal**	2a) Relationships with partners can have a substantial influence on ART initiation, adherence, and retention.	**High** This finding was reported across 14 studies, with quantitative and qualitative designs. Five of the papers also reported on issues around gender dynamics and six of the papers reported on the benefits of disclosure to a partner to adherence. One quantitative paper reported a significant and contrary finding that not disclosing HIV status to a partner enabled adherence, but this was not reported elsewhere.	**High** This broad finding was reported across a wide range of contexts, arguing for a high validity. The one finding that non-disclosure to a partner enabled adherence highlights how the nature of interpersonal influence may vary by context.
	2b) Relationships within the family affect ART initiation, adherence, and retention.	**Moderate** This finding was reported in three qualitative papers and a mixed method study. Designs were robust and the evidence was descriptive in all four studies.	**Low** This finding was reported in a small number of largely descriptive papers.
**Community**	3) Stigma within a community can be a significant barrier to ART initiation, adherence, and retention.	**High** This finding was reported in 15 papers, almost all of which were based on qualitative studies. Data on stigma as a barrier typically came in the form of participant self-report. One quantitative study described self-disclosure as an enabler of access to HIV care and adherence to ART.	**High** This finding was reported across a wide range of contexts, especially studies in which women identified barriers to initiating, adhering to, or remaining on treatment.
**Structural**	4a) Higher participation in recommended health services leads to increased likelihood of ART initiation, adherence, and retention.	**Moderate** This was a core finding in six quantitative papers. Delivery in a health center was found to be an enabler in two studies, although one was derived from a conference abstract with limited detail. Two papers reported an association between ART adherence and receiving treatment (e.g., tuberculosis treatment or multivitamins) for other conditions.	**Moderate** This finding was reported across a range of contexts.
	4b) Logistical problems around access to services can be barriers to ART initiation, adherence, and retention.	**Moderate** Seven papers with a range of designs (i.e., four qualitative, two mixed method, and one quantitative) reported this finding. Transportation problems were described descriptively in four well-designed studies, and cost was reported as a limited finding in one quantitative study. Long queues at health facilities were report as barriers to initiation, adherence, and retention in four papers of differing methods.	**Moderate** This finding was reported across a range of contexts and designs. The finding is generalizable, though specific factors may be context-dependent.
	4c) Interactions with health workers are valued, and affect the quality of access, and likelihood of ART initiation, adherence, and retention.	**High** Seven studies reported that health worker attitudes influenced whether women initiated and adhered to ART. Qualitative studies with strong designs reported that women’s perspective of beneficial interactions encouraging adherence. One quantitative paper proposed a second order interpretation that adherence is due to nurses taking opportunities to engage more effectively with patients. Positive interactions with doctors and traditional birth attendants were noted as enablers of ART initiation, adherence, and retention in two strong qualitative papers.	**Moderate** This finding was reported qualitatively across a range of contexts, suggesting strong context validity, and reasonable generalizability.

The findings extracted from the studies were identified with respect to which outcomes they related to (initiation, adherence, or retention), which region of the world they came from, the socioeconomic status of study participants, and whether they were from low or high prevalence settings. We used these characteristics to develop ‘sub-groups’ of the various findings, searching for example, for whether or not there were distinct variations in the barriers and enablers identified by region, or by type of outcome. In Table 3, and in the narrative findings below, we distinguish which specific findings applied to each of the three outcomes. We also make consistent reference in the narrative to the countries and world region(s) that contributed to particular findings.

Making further generalizations about patterns of findings within these sub-groups has been difficult, however, for several reasons. One is that the body of studies that contributed the most to the review findings are the studies that came from high-prevalence settings in Eastern and Southern Africa. A second challenge has been the fact that in most cases, findings are only supported by one, two, or three studies, making comparisons along various axes of region, prevalence, or outcome difficult. Finally, many of the studies provide little detailed context on the study setting and implementation process of the programs under review. Absent such context, it is difficult to develop confident explanations about how context (e.g. HIV prevalence or the SES of participants or the wealth of a particular country) might affect the finding in more precise ways.

### Individual factors

Individual-level enablers and barriers to pregnant or postpartum women’s ART initiation, adherence, and retention include those within a woman’s awareness and control (e.g., commitment to a child’s health), and those that may be outside of her awareness or control (e.g., where she lives or her level of knowledge about HIV, ART, or PMTCT).

#### Socio-demographic attributes

Age was associated with ART initiation, adherence and retention in several of the included studies, albeit in contrasting ways. Two studies, one from Tanzania and one from the US, found that younger women were less likely than older women to engage with the health system and/or adhere to ART [25], [35]. In contrast, a multi-country study of long-term ART in Latin America found that non-adherence increased by six percent with each one-year increase in age. The study’s authors posited that these findings might relate to the higher demands on older women living with HIV, particularly those with children under 18 [37].

Education was also noted as an important factor. Two studies from Kenya and one from the US found that women’s education level was positively associated with ART adherence [20], [21], [36]. Ayuo et al. [20], in their Kenyan study of pregnant women initiating ART, reported that each additional year in school increased the likelihood of reporting perfect adherence by 10.6 percent. Similarly, a study in Rwanda found that women with lower education levels were less likely to participate in an sdNVP program. The study’s authors posited that higher education levels contributed to better health literacy, which in turn promoted sdNVP program initiation and adherence [26].

Finally, two studies in Kenya found that rural residency was a barrier to ART initiation and adherence. In the first study, women enrolled in HIV care at a rural clinic were more likely to be lost to follow-up than women enrolled in similar care in a district hospital [20]. In the second study, a qualitative narrative analysis, HIV-infected pregnant women in rural settings were less likely to disclose their HIV status than urban women. The authors argued that these women, in striving to keep their status a secret, were more likely to miss clinic appointments, resulting in poor ART adherence [48].

#### Knowledge of HIV, ART, PMTCT and the Health Services

In three studies in Uganda, South Africa, and Tanzania, a lack of knowledge about health services and/or ART was associated with poor ART adherence [41] or retention [27], [50]. Similarly, three other studies in Kenya, Ghana, and South Africa found that sufficient knowledge of PMTCT facilitated ART initiation, adherence and/or retention during and after pregnancy [22], [36], [47]. For example, the Ghana study found that many study participants had a high level of essential HIV knowledge (e.g., routes of transmission; the role of ARVs in prolonging life), but that women with inadequate knowledge of PMTCT and ART were significantly more likely to be lost to follow-up [22].

#### Fears and Aspirations Related to HIV, ART and Motherhood

Several studies identified women’s fears as barriers to ART initiation and/or adherence. For example, fear of losing a job or fear of being HIV-infected contributed to inaction and/or denial about one’s HIV status. Some studies found that women feared HIV testing or the ARVs themselves, including a South African sdNVP study [47] and studies on ART in Tanzania and Malawi [23], [50]. Two South African studies found women’s unwillingness to commit to lifelong treatment as a barrier to ART initiation [43], [47]. Furthermore, two studies from the US and Australia found that women feared ART would have a negative impact on their children [39], [40].

The review of literature also found that women’s desire to protect her health and her children’s health could positively influence ART initiation, adherence, and retention. In a Nigerian study, an active desire to remain healthy and/or to protect one’s child was an enabler of ART initiation and adherence [28]. A desire to protect one’s children also motivated ART adherence among women in a US study [40] and ART initiation among women in two South African studies [43], [47].

Women’s concerns about maintaining their status within their families sometimes led them to keep their HIV infection a secret, creating a barrier to initiation, adherence and retention. Some women in a Kenyan study felt that disclosure of their HIV status would undermine their roles as mothers and homemakers. This non-disclosure made ART initiation and adherence particularly challenging for the women during pregnancy, when elder women in their families make decisions about their health care [48]. Another Kenyan study found that women were reluctant to attend clinics for ART services because their visibility during long waiting times could reveal their HIV status and, in turn, enhance their risk of being stigmatized and perceived as incapable mothers [19].

#### Challenges to the Practical Demands of Treatment

Individuals experienced a wide variety of barriers to the day-to-day practical requirements of treatment adherence. These included difficulty remembering to take medication, misplacing medications, travel away from home, scheduling conflicts with work, and lack of regular access to food or water. Forgetting to take medication was reported in six studies, including an sdNVP study in Rwanda [26], and pregnancy and postpartum ART studies in Latin America, Nigeria, South Africa, and Tanzania [28], [35], [37], [41]. Research in Zimbabwe found that being away from home and/or misplacing medicines impeded women’s ART adherence [38], and similarly, a study in South Africa found that being away from home created a barrier when treatment was required [41].

Women also reported that scheduling challenges (e.g., due to work commitments) affected both adherence and retention, specifically for registration or ART visits in Nigeria and Zimbabwe and for support group meetings in South Africa [28], [42], [52]. Three studies in Kenya, Tanzania, and South Africa identified lack of food, water, or income as barriers to ART adherence; women were less likely to adhere to their medications when food was unavailable, because taking ART on an empty stomach often caused negative side effects [19], [35], [41].

Two studies in Africa, on the other hand, found that ART adherence among women was facilitated by the use of mobile phones and/or enrolment in a postnatal care program. Women in a South African study said they forgot to take ART medication because their phones were turned off, which the authors interpreted to mean that the women were using their phones for personal organization [41]. In Uganda, women were three times more likely to attend a six-week postnatal care appointment if they had provided a phone contact. The study’s authors posited that such women were more open to being contacted by health personnel than women who did not provide a phone contact, and also might be more likely to accept entry into the medical care system and/or to have a good socioeconomic status that empowered them to make informed decisions [44].

#### Religious Beliefs

Religion was found to influence adherence and retention in three studies. In a quantitative study in Uganda, being Christian was found to be a predictor (through correlation) for ART adherence among women over 25 years of age [44]. Another quantitative study in Zimbabwe found that belonging to a religion that promoted the use of traditional herbs during pregnancy (i.e., not biomedical care) reduced visits to antenatal care (ANC) clinics and/or use of sdNVP [38]. Similarly, a mixed-method study from Ghana found that the use of alternative medicines and/or participation in overnight prayer camps contributed to ART interruption and loss to follow-up [22].

#### Alcohol and Substance Abuse

Alcohol and/or illicit drug use were barriers to ART adherence during and after pregnancy in two U.S. studies [21], [25], and a multi-country Latin American study found tobacco use was negatively associated with adherence [37]. No studies from sub-Saharan Africa included these factors in their data.

### Interpersonal Factors

Interpersonal barriers and enablers are those influenced by both the woman and other individuals in her life, such as her partner or family members. The review found that a woman’s relationships within her immediate family could profoundly influence her ART initiation, adherence and retention. The degree of the impact of these factors often depended on the extent to which she had disclosed her status and the extent to which family members who knew her status supported her. Interpersonal findings specific to women’s partners or spouses are discussed below, followed by findings related to other family members.

#### Spouse or partner

Eighteen studies highlighted the role and impact of a spouse/partner on ART initiation, adherence, and/or retention. This was one of the most widely reported findings across the reviewed studies. Studies in Rwanda, Uganda, and Malawi found that women often felt a need for their partners’ permission to initiate, adhere to, and be retained in ART care [26], [27], [45]. In a rural Tanzanian study, women reported being dependent on their partners for transportation to health facilities, so consistent participation in an ART program was difficult if they had not disclosed their status [35]. Similarly, in Uganda, non-disclosure of HIV status to a partner was the second most commonly cited barrier to enrolling in a PMTCT program [27].

Many of these women explained that economic dependence on their husbands and/or fear of domestic violence inhibited them from disclosing their status [27]. Domestic violence - actual or anticipated - was also reported as a barrier to disclosure and ART adherence in a South African study [41].

Other studies in France, Kenya, South Africa, and Tanzania also found that non-disclosure of HIV infection to a spouse was a barrier to ART initiation and retention [29], [30], [47], [50]. Three of these studies were qualitative and provided detailed insight into women’s relationships with their husbands, and the influence of those relationships on ART interventions. For example, in the South African study, some women specifically asked to delay their ART enrolment because they needed more time (e.g., a week or a month) to disclose their HIV status to their partners [47]. Two quantitative studies in Zimbabwe and South Africa found strong associations between a woman disclosing her HIV status to a partner and taking sdNVP, as recommended, at the start of labor [38], [46].

Several studies assessed the impact of a partner’s involvement in a woman’s HIV care. Partner involvement was indicated by factors such as knowledge that the woman had been referred to HIV treatment, accompanying her to health appointments, or participating in couples counselling. In Kenya and Zambia, studies identified partner involvement as an enabler of ART initiation. Similarly, in South Africa, partner involvement was identified as an enabler of ART adherence [32], [36], [46]. Findings for this factor in relation to retention were inconsistent in Malawi, however, where one study found lack of male involvement as a barrier to ART retention [23], and another found it as an enabler [34].

#### Family

Family members were cited as either important facilitating or inhibiting influences by different studies. A family’s embrace of community norms around stigma (discussed in more detail below as a key community-level factor), and the consequent pressure to maintain role and status within families can lead women to keep HIV infection a secret, creating a barrier to ART initiation and adherence. This relates to the barrier created when decision making about the pregnant woman’s health/health care is made by an elder female family member, as seen in a Kenyan study [48]. Another study from Malawi found that grandmothers strongly influenced pregnant women’s choice of delivery, provider and location options (e.g., a traditional birth attendant at home) [45]. This lack of autonomous maternal decision-making capacity appeared to restrict HIV-infected women’s ability to adhere to ART and PMTCT protocols. Only one study reported broader family support as an enabler of adherence during pregnancy, by helping women more with domestic tasks at home and allowing them time to attend appointments [60].

#### Community Factors

This section addresses barriers and enablers of pregnant and postpartum women’s ART initiation, adherence and retention at the community level. These factors relate to a woman’s broader social network and context. Stigma and disclosure are the two critical community-level factors that appeared most frequently in the studies described below.

#### Stigma

Like spousal disclosure and support at the interpersonal level, stigma was one of the most widely reported influences on women’s ART initiation, adherence and retention at the community level. In some cases, women’s direct experience of stigma were barriers to successful ART outcomes, while in others, fear of anticipated stigma was a barrier for women who had not disclosed their HIV status publicly. Seven qualitative studies, two mixed-methods studies, and two quantitative studies reported stigma as a barrier.

Studies in Uganda, Malawi (two), South Africa, and Tanzania found that HIV-related stigma was a barrier to women’s ART initiation and retention, or their use of sdNVP. Some women in these studies reported not initiating treatment because they feared others would learn of their HIV infection and blame or stigmatize them; some also feared their husbands would divorce them [23], [45], [49], [50], [61].

Similarly, other studies in Malawi, South Africa (three), Nigeria, Uganda, and Kenya reported that stigma inhibited ART adherence and, in one case, retention. Many women who had not disclosed their HIV status publicly feared there was insufficient confidentiality within health facilities; this fear contributed to women missing appointments and not participating in broader HIV services, such as patient support groups [24], [28], [41], [43], [44], [48], [52].

#### Disclosure

Studies in Zimbabwe and South Africa identified positive experiences with disclosure as enablers of ART adherence during pregnancy [38], [46]. Specifically, women in the Zimbabwe study reported that disclosure to someone other than their spouse had been beneficial to their adherence [38]. The South African study similarly found that disclosure not resulting in stigmatization was positively associated with maternal ART adherence. A third study from Kenya found that having ART clinics separate from main hospital buildings reduced clinic attendance and ART adherence; women were concerned their HIV infection would be disclosed publicly by their attendance at the HIV-only sites [60].

#### Structural factors

Structural influences on a woman’s ART initiation, adherence and retention are those within her broader environment (beyond the local community/social context) that are beyond her control and agency (e.g., organizational, economic, legal, and policy factors). The main structural barriers and enablers identified in this review relate to health system usage, access, and engagement. The studied reviewed rarely made mention of broader other barriers such as economic marginalization, gender norms or the legal and policy context. Most reference to these factors were made in relation to issues of health care access and use.

#### Access to health services

Difficulty obtaining or paying for transport to facilities was a barrier in studies from Uganda, Tanzania, Malawi, and South Africa [27], [35], [45], [47], [50]. No studies specifically highlighted the costs of services as a barrier to ART initiation, adherence or retention. However, authors of a Kenyan study speculated that the cost of HIV service registration may have contributed to some women not attending services or being lost to follow-up [29], while authors of a Nigerian study posited that the free nature of services was an enabler of adherence [28]. Additionally, several studies identified long queues and wait times as barriers to ART initiation, adherence, and retention in Kenya, Malawi, and Uganda, and Zimbabwe [24], [27], [42], [60].

#### Use of health services

Several studies found that the more women participated in recommended health services, the more likely they were to initiate, adhere to and be retained in ART care. For example, studies in Tanzania and Kenya found that low antenatal care (ANC) attendance (i.e., less than three visits) was associated with lower rates of ART initiation and retention [29], [50]. Interestingly, women who were pregnant for the first time were more likely to register at an HIV clinic than women who had been pregnant before–the authors speculated this might be because women tended to be more anxious about their own and the fetus’ health during their first pregnancy [29]. In a Kenyan study, women who disengaged from HIV services in the 30 days before delivery (i.e. late disengagement) were more likely to be lost to follow up postpartum when compared to women who stayed until delivery [20].

Delivery at a health facility was associated with ART initiation in studies in Kenya and Rwanda [26], [36]. The Rwandan study found that women delivering in health centers were more likely to have had two or more ANC visits, and to have received sdNVP at the onset of labor [26]. Although this study focused on sdNVP use, it was included in this review because it is indicative of the enabling effect of prior interaction with the health system. In the Kenyan study, women receiving treatment for other conditions (e.g., tuberculosis) were less likely to disengage from ART services, and those receiving PMTCT were more likely to register at an HIV clinic and to be retained in long-term HIV care [20].

#### Health worker attitudes

Eight studies reported that health workers’ attitudes influenced women’s initiation and adherence to ART. Studies in Brazil, Kenya, Malawi and South Africa found that negative health worker attitudes were barriers to ART initiation [29], [31], [33], [49]. Studies from Australia, Malawi, Uganda, and South Africa found that negative attitudes were a barrier to both adherence and retention during and/or after pregnancy [27], [39], [45], [47], [49]. In these studies, negative provider attitudes were exemplified by health workers in Uganda who reportedly were uninterested or too busy to interact with women or provide them with medication [27], and by health workers in Malawi who reportedly shouted at women attending HIV services [45]. Studies in Kenya and Malawi found that women’s concern that health workers would not maintain confidentiality also inhibited adherence or retention [24], [60].

Positive, non-judgmental attitudes from health workers – described in a Brazilian study as “warmth” – were found to be an enabler for ART adherence [31]. A Malawian study found that traditional birth attendants (TBAs) were preferred over health workers because they were more accessible and positive towards women, underscoring the importance of ensuring respectful care in antenatal, maternity, postnatal and HIV services [45].

## Discussion

### Overview of Findings and Programmatic Implications

This review identified a range of individual, interpersonal, community, and structural factors that inhibit or facilitate HIV-infected pregnant and postpartum women’s ART initiation and adherence. Only four of the 34 included studies described or evaluated an intervention. Thus, we are not able to make evidence-based recommendations for specific interventions to improve HIV-infected women’s access to and use of prenatal and postpartum ART. However, the review’s descriptive findings suggest broad areas of intervention needs for this population, and these are discussed below in the context of the general literature on ART adherence barriers, facilitators, and interventions.

#### Individual level factors

This review found that many studies reported barriers to ART initiation, adherence, and retention that were related to poor understanding of HIV, ART, and PMTCT. For example, some women believed they did not need to initiate ART because they felt too healthy or feared ART would harm the fetus. Other studies identified poor knowledge about how PMTCT worked as both a barrier to ART initiation and adherence as well as retention in care postpartum. These findings may be especially important going forward with the growing roll-out of Option B+, as increasing numbers of healthy-feeling women are initiated on ART [62].

This review did not set out to assess women’s knowledge of HIV, ART and PMTCT and can therefore make no general findings about these levels of knowledge. The review can provide indirect evidence, however, that gaps in knowledge about HIV, ART and PMTCT continue to act as barriers to critical health services and highlight the need for improving the provision of information within and outside of health services. While it is disappointing that poor understanding of HIV still persists, this finding indicates a promising way forward, in that improving knowledge is generally considered one of the simplest and most straight-forward behavior change interventions [63].

Other common individual-level problems were forgetting to take ART, misplacing it, or not having access to it when traveling. Our findings indicate the women would benefit from support in developing routines and approaches for self-monitoring and remembering to take their medication. Such interventions should use locally available and culturally appropriate systems, such as intensive counseling within health facilities or strengthening community-based support systems (e.g., maximizing the discreet and convenient potential of mobile-phone text reminders or supporting home-based care providers who remind women to take and renew their medications and accompany them to appointments, as needed) [64]–[67]. The discipline, commitment, and coordination required for ART adherence over an extended period should not be underestimated.

#### Interpersonal level factors

Many studies reported on the crucial influence of husbands or partners, both positive and negative. Disclosure of HIV infection to a spouse and spousal involvement in a woman’s treatment were both associated with improved initiation, adherence, and retention. However, multiple studies also reported that women were reluctant to disclose their HIV status to partners because they feared significant negative consequences. Importantly, it is not possible to know the direction of cause and effect in these findings. It is possible that women who choose to disclose to their partners already have positive and supportive relationships, in comparison to women who do not disclose, and that these positive relationships in themselves promote ART initiation, adherence, and retention. Alternatively, for some women, the process of disclosure itself (regardless of relationship quality) may be a critical factor in promoting ART initiation and/or adherence.

In either case, these findings highlight the importance of ART programs that focus on the continuum of care for women, acknowledge the role these relationships may have in ART initiation, adherence, and retention, and incorporate interventions that take into account the relevance of women’s primary relationships. Maternal ART program staff can provide counseling for women who have not yet disclosed their status to their partners, communication skills exercises, couple counseling to help them to disclose, or practical strategies for ART adherence if they are unwilling or unable to disclose to their partners. Very few of the interventions identified in this review addressed the role of power and interpersonal relationships, reflecting a broader tendency among individual-level HIV interventions to focus on knowledge and planning skills [68].

#### Community level factors

At the community level, this review found the experience of stigma, or the fear of stigma, to be a substantial barrier to ART initiation, adherence and retention among pregnant and postpartum women. Other reviews have also found stigma to have a significant influence on ART initiation and adherence [69]. Basic misunderstandings about HIV and AIDS persist in much of the world; for example, UNAIDS’ *2010 Global Report on the AIDS Epidemic* found that, in 15 of the 25 countries with the highest HIV prevalence rates, less than half of young people could answer five basic questions about HIV correctly [70]. Misunderstandings about the effectiveness and value of ART and PMTCT may contribute to community-level stigmatization of women who are discovered to be HIV-infected because they participate in ART programs. These findings highlight the need for intensive interventions focused on raising knowledge and awareness about the effectiveness and value of ART and PMTCT, and the harmful effects of stigma [71], [72].

#### Structural level factors

This review’s findings underscore the importance of HIV services providing intensive, targeted support tailored to the unique needs and circumstances of HIV-infected women during pregnancy. For effective ART initiation, adherence, and retention, women may need in-depth orientation and counseling sessions to ensure they fully comprehend the importance of ART, to help them through the process of disclosing their status to husbands/partners/families, and to strategize about how they can reliably take their medication [73], [74].

HIV services ideally should be integrated with other maternal and child health services to maximize efficiency for the client, service delivery, and confidentiality (e.g., one-stop ANC and ART appointments). Integrated services would also support confidentiality and reduce fear of stigma among women who have not yet disclosed their status. Regardless of the specific program design of the maternal, child, and HIV health services in a particular context, however, this review also highlighted the critical role that health worker attitudes played in encouraging–or more often discouraging–access to and use of HIV services.

### Are the pregnancy and postpartum periods unique?

Many of our findings related to ART initiation, adherence, and retention for HIV-infected pregnant and postpartum women are similar to those reported for people living with HIV (PLHIV) more broadly, including enablers such as social support [64], [65] and barriers such as fear of disclosure and stigma, lack of knowledge about HIV and ART, or difficulty obtaining transportation to facilities [74], [75]. In fact, the most urgent findings from this review–the continuing influence of stigma at both interpersonal and community levels, the surprising persistence in knowledge gaps, and the ongoing missteps and missed opportunities in the HIV-related health services–reflect long-standing challenges in ART programming for adults more generally.

A question arising from this recognition, however, is how individual and contextual barriers and enablers for pregnant and postpartum women might differ in important ways from those of the general population of PLHIV. While we did not directly compare findings between pregnant and non-pregnant women with HIV, the review suggests that some enablers may have a stronger influence on pregnant and postpartum women. For example, pregnant women are more likely than others to visit health facilities regularly, which, under ideal circumstances, would promote ART initiation, adherence, and retention and relatively prompt care for other conditions. A recent assessment of the beneficial effect of pregnancy on presentation for HIV care found that non-pregnant women were twice as likely to present late when compared to pregnant women [76]. The studies in this review also identified ways in which pregnant women may receive special support from partners and other family members who assist them in domestic and other work activities. Concern for the health of the fetus may also facilitate or inhibit adherence to ART.

Some barriers to ART initiation, adherence, and retention may also be intensified for pregnant or postpartum women. Women in multiple studies reviewed reported increased demands or responsibilities due to their pregnancy, caring for an infant, and/or physical conditions post-delivery as barriers to adherence or retention in care [19], [42], [45], [47]. Many women maintain their usual responsibilities with little assistance while pregnant. Pregnancy can be physically demanding and tiring, and there may be additional commitments to maintain, including regular health care appointments at distant facilities. This highlights a particular concern for pregnant women who only learn of their HIV infection when screened during pregnancy, and are then initiated on ART. These women must quickly adjust to being pregnant, being HIV-infected, and to a daily treatment regimen they will need to follow for the rest of their lives. Each of these changes may tax a woman emotionally and physically, and may be further exacerbated during the postpartum period, when she is both recovering from the delivery as well as breastfeeding and caring for an infant day and night. All of these conditions help suggest why barriers to ART initiation, adherence, and retention, common to many PLHIV, may become more pronounced for some women during pregnancy and postpartum.

### Review limitations

The strengths of this review’s design included its inclusive search strategy that ensured wide coverage, dual inclusion and data extraction, and iterative analysis. Limitations in the review design included its rapid pace, which prevented more exhaustive searches of the gray literature and inclusion of studies in languages other than English.

Given the diversity of the evidence base with respect to settings, ART/PMTCT program types and protocols, and study questions and designs, we have not been able to transform or interpret the data beyond the descriptive, thematic analysis presented above.

The diversity of evidence included here is a strength in terms of the richness it provides, but generalizability and transferability across contexts was limited by a range of factors. First, there was great variance in how adherence was defined and measured across the reviewed studies, limiting our ability to synthesize findings across studies (see Supporting Information for a table summarizing the outcome measures used in the included studies). Second, studies differed greatly in the range of ART regimens they evaluated, which also limited our ability to integrate and interpret findings, particularly since some ART barriers and enablers may be particular to specific regimens.

Third, many studies did not clearly distinguish between individual and contextual factors influencing ART initiation, adherence, and retention *during* pregnancy from those influencing *postpartum* ART adherence, a time when conditions for women are likely to be very different. Finally, many identified barriers and enablers may be regionally or culturally specific. While this may in general limit the transferability of the findings outside of sub-Saharan Africa–and in particular high-prevalence regions within Southern Africa–it does potentially increase their transferability with this region, and possibly to regions with similar levels of HIV prevalence and health system challenges. A further threat to transferability of the contextually specific findings, however, is the fact that few of the articles provided rich enough detail to know when particular findings might be transferable to similar contexts.

### Research agenda

This review has revealed several gaps in the existing evidence base, gaps that collectively point to what we argue should be key parts of the research agenda going forward. Perhaps the most important gap, and a critical focus of future research, is the women who do not appear in these studies–those who did not make it to antenatal care or HIV testing, or who dropped out along the maternal ART cascade. Much of the research we reviewed is focused on health systems issues and women’s engagement with these systems. Consequently, we know a great deal more about who stayed in care and why than about those who never attended entered the system or who dropped out early [77]. None of the included studies examined the barriers that women who do not enter into care experience in accessing maternity services and initiating ART during pregnancy or postpartum. We need a great deal more research–and programming–aimed at understanding and supporting such women. Loss-to-follow-up is particularly likely to happen postpartum [40], which suggests this is an area that also needs increased attention as Option B+ is scaled up.

There is also limited examination of health beliefs in the literature. Alternative treatment-seeking, particularly for traditional medicine, is widely practiced in many countries with high HIV prevalence [78], but these choices were minimally addressed within the reviewed studies. This likely reflects the number of studies that took place within the health system, and highlights the need for community-based research focused on how alternative health-seeking behaviors may intersect with ART initiation, adherence, and retention among pregnant and postpartum women. Similarly, our understanding of how interpersonal and community-level factors operate would benefit from more research examining the impact of HIV and treatment on women’s aspirations and expectations, their roles and status within families, and the broader social dynamics in relation to pregnancy and HIV.

Finally, this review highlighted a critical methodological gap and area for future improvement–the need for meaningful, standardized ART adherence and retention performance indicators within programs and research. Measuring ART adherence and retention is critical for PLHIV care and treatment and broader efforts to minimize ART resistance, but collecting valid data on these outcomes is not straightforward. Adherence measures based on dispensing and appointment-keeping data are increasingly being implemented within health systems [79], but measures which assess adherence behaviors more broadly among PLHIV vary widely. There are also important questions to answer, however, with respect to how valid adherence measures for pregnant and postpartum women may differ from standard appointment-based measures in the short-term, given pregnant women’s unique schedule of contact with the health system during pregnancy and postpartum. Finally, very few of the studies measured adherence or retention measures in the long-term and provided little guidance on how and why women may cycle through periods of better and worse adherence or even drop in and out of care episodically. Our understanding of pregnant and postpartum women’s access to and use of ART would benefit greatly from research that used consistent, standardized, and appropriate measures of adherence and retention that had a longitudinal component.

## Conclusion

The potential of antiretroviral therapy to prevent avoidable maternal deaths among HIV-infected women is great. The success of this strategy, however, will depend on careful consideration of the barriers and enablers to pregnant and postpartum women’s access to and use of ART. Managing these barriers and enhancing known enablers will require the development of respectful and locally acceptable HIV and ANC service delivery models that are responsive to women’s needs and perspectives, and support them as they enter and move through the maternal ART cascade. It will also require better understanding the ways women’s lives outside of the clinic affect their chances of entering into, and staying adherent to and retained in care.

This review of individual and contextual factors, along with the two companion reviews which assess the evidence on the effectiveness of interventions to decrease death and morbidity among HIV-infected pregnant and postpartum women [12] and examine the health system barriers and enablers to ART initiation, adherence, and retention among this group [13], point to strategies that may be effective in expanding the reach of ART. Translating this evidence into practice is critical for keeping pregnant women and mothers alive and preventing new HIV infections among their children as we endeavor to achieve the global goals of an AIDS-Free generation and ending preventable child and maternal deaths.

## Supporting Information

Table S1 **Search Strategies.**
(DOCX)

Table S2 **Characteristics of Included Studies.**
(DOCX)

Table S3 **Findings by Level, Theme, and Study.**
(DOCX)

Table S4 **Outcome Measures Used in the Studies.**
(DOCX)

Checklist S1 **PRISMA Checklist.**
(DOCX)
