# Supplementary material for: A Systematic Review of Individual and Contextual Factors Affecting ART Initiation, Adherence, and Retention for HIV-Infected Pregnant and Postpartum Women
Source: PLoS One. 2014 Nov 5;9(11):e111421. doi: 10.1371/journal.pone.0111421 (PMC4221025; doi:10.1371/journal.pone.0111421)
Supplement: Table S1 — Search Strategies. (DOCX) [file pone.0111421.s001.docx]

# Search Strategies

**Full search strategy for PubMed and Social Sciences Citation Index**

((((pregnant OR partum OR natal OR breastfeed* OR mother OR maternal)) AND HIV) AND (ARV OR ART OR haart OR pmtct OR antiretrovir* OR treatment)) AND (retention OR adherence OR compliance OR initiation OR ltfu OR loss to follow up))

Dates: 2008 (inclusive) to present

**List of Gray Literature Sources Reviewed**

| **Source** | **Number of Documents Screened** |
| --- | --- |
| United Nations Joint Program on HIV/AIDS (UNAIDS) | 142 |
| World Health Organization (WHO) | 224 |
| United States Agency for International Development (USAID) – Development Experience Clearinghouse (DEC) | 303 |
| ICAP, Columbia University | 26 |
| Elizabeth Glaser Pediatric AIDS Foundation (EGPAF) | 66 |
| Pathfinder International | 10 |
| International AIDS Society (IAS) | 620+ |
| Conference on Retroviruses and Opportunistic Infections (CROI) | 100+ |
| International Society For Sexually Transmitted Diseases Research (ISSTDR) | 95 |
| International Conferences on Improving Use of Medicines (ICIUM) | 50+ |
