# Supplementary material for: A Systematic Review of Individual and Contextual Factors Affecting ART Initiation, Adherence, and Retention for HIV-Infected Pregnant and Postpartum Women
Source: PLoS One. 2014 Nov 5;9(11):e111421. doi: 10.1371/journal.pone.0111421 (PMC4221025; doi:10.1371/journal.pone.0111421)
Supplement: Table S2 — Characteristics of Included Studies. (DOCX) [file pone.0111421.s002.docx]

# Characteristics of Included Studies (Full Table)

| **No.** | **First Author (Publication Year)** | **Location** | **Design^[[1]](#footnote-1)^** | **Population Size** | **Type of ART Program** | **HIV Prevalence** | **Socio-demographic characteristics** | **Key Findings about ART Enablers and Barriers** |
| --- | --- | --- | --- | --- | --- | --- | --- | --- |
| 1 | Awiti-Ujiji (2011) | Kenya | QL | 26 | Standard ART | Slightly higher than national average (both locations); national prevalence rate for gen pop is 6.4%; 8% for women (DHS 2008/09) | Low (Over half of the pop living in povery in Kibera; average household income $84USD per month in Busia); Kenya is classified as a low-income country according to World Bank | The main barrier to ART adherence was a conflict between women's responsibilities as mothers and their needs as patients, particularly postpartum, when family members did not understand or support women attending medical appointments. |
| 2 | Ayuo (2013) | Kenya | QT | 4,284 | Option A | National prevalence rate for gen pop is 6.4%; 8% for women (DHS 2008/09) | Low; Only 11.6% employed outside the home; Median 8 years of education; Kenya is a low-income country according to World Bank | Imperfect adherence was associated with lower-level health facilities, rural settings, and fewer years of education. |
| 3 | Aziz (2011) | Uganda | QT | 40 | Option A | Prevalence estimated at 30% in this setting, according to article (unclear if referring to adult prev or among preg women) | Abstract did not report on socio-dem factors; Uganda is low-income according to World Bank | The main enabler of ART initiation was availability and experience of counseling. |
| 4 | Bardeguez (2008) | U.S. | QT | 519 | Option A;  Option B;  Standard ART | National prevalence rate for gen pop is 0.6% (2009) | Study in US (high income country by World Bank classification); Only 17.7% had beyond high school education level | Respondents were most likely to report perfect adherence on visits shortly before delivery; they were least likely to report this postpartum. Factors significantly associated with perfect self-reported adherence were: initiation of ARVs during current pregnancy; less-advanced HIV-1 disease; no use of marijuana; feeling happy all or most of the time; and never missing prenatal vitamins. |
| 5 | Boateng (2013) | Ghana | MM | 203+23 | PMTCT prophylaxis (regimen not stated);  Standard ART | 3.9%; higher in urban than rural | 70% employed and earn less than $105.46 per month; 21% no formal education; Ghana is lower-middle income according to World Bank | Rates of HIV program default (i.e., consistently missing two or more appointments in the previous two months) were higher among women with inadequate knowledge of PMTCT and ART. Older age was associated with adequate knowledge of PMTCT and ART. |
| 6 | Bwirire (2008) | Malawi | QL | 16+9 | sdNVP | Adult prevalence rate 14% and antenatal prevalence 15% (in article, MOH 2005) | Does not provide indicators but would consider it Low based on barriers cited (cost of transport, shortage of trained health staff, need to decentralize health services to reduce waiting times) | Barriers to ART initiation were: women feeling that HIV testing was obligatory; women not being prepared for their test result; fear of stigma upon disclosure; partners not wanting to be tested, and partners not being supportive. Long waiting lines at ANC clinics were the main barrier to ART adherence during pregnancy. |
| 7 | Chinkonde (2009) | Malawi | QL | 28 | Option A | Prevalence among pregnant women 15% (in article) | Variety - sites specifically selected to reflect difference socio-economic profiles; 39% were illiterate; only 7% were in informal employment outside the home | Barriers included a spouse's refusal to be tested or to disclose HIV status; long clinic waiting times; negative health worker attitudes; and women's concern that participation in an ART program (particularly home visits and attending PMTCT sites close to ANC clinics) would unintentionally disclose their HIV status. The main enabler of ART adherence was a spouse's positive support. |
| 8 | Cohn (2008) | U.S. | QT | 149 | Standard ART | National prevalence rate for gen pop is 0.6% (2009) | Study in US (high income country by World Bank classification); 38% had less than high school education | Illicit drug use and missing prenatal vitamins were associated with lower adherence to ART pre- and postpartum. |
| 9 | Dean (2012) | South Africa | QT | 7 [1,018 texts] | Option A  Option B | 30% prevalence among pregnant women in South Africa (in article) | Women were recruited in townships in Pretoria so assume Low; All could read and write in English and were able to communicate via text message; Education level varied from completion of grade seven to high school; one of seven women was employed full time; South Africa is classified as upper-middle income by World Bank | Use of cell phone texts for medical information and psychosocial dialogue were ART enablers. |
| 10 | Delvaux (2009) | Rwanda | QT | 236+125 | sdNVP | National prevalence rate for gen pop is 3%; 3.6% for women (DHS 2005) | Both (higher and lower SES determined by having lighting and ownership of a functioning radio); Rwanda is a low-income country according to World Bank | Non-adherence was associated with fewer ANC visits, less trust of clinic staff, and lower disclosure rates to partners, family members, and friends. Waiting for spousal permission and forgetting to take pills were also cited as initiation and adherence barriers. |
| 11 | Duff (2010) | Uganda | QL | 45 | sdNVP  Option A | Adult prevalence rate 5.4%; 7.5% among women (in article) | Low SES (compared to Ugandan counterparts); Uganda is low-income according to World Bank | Barriers were organized in the following five categories in order of frequency cited: economic factors, social/environmental factors, health care factors, HIV/HAART knowledge, and HIV disease progression. |
| 12 | Ekama (2012) | Nigeria | MM | 170 | sdNVP  Standard ART | Adult prevalence rate 3.5% (UNAIDS 2009) | 76.5% of women were employed/working; 81.1% had at least secondary education; Nigeria is lower-middle income country according to World Bank | The main adherence enablers were women's desires to remain healthy and alive and to protect their unborn children from HIV infection. The main barriers to adherence were forgetfulness, demanding work schedules, and fear of being identified as HIV-infected. |
| 13 | Ferguson (2012) | Kenya | QT | 1,129 | PMTCT prophylaxis (regimen not stated)  Standard ART | 8.8% prevalence among women (in article) | Unclear; only captured data on age, year of first hospital visit, marital status, # of pregnancies, gestational age, distance from hospital, and MCH register where woman first appeared; Kenya is a low-income country according to World Bank | Registration at an HIV clinic was higher for women in their first pregnancies and for women complying with scheduled ANC visits. |
| 14 | Jasserson (2013) | France | QT | 2,952 | Option A | Adult prevalence rate 0.4% (UNAIDS 2012) | Study in France (high income country by World Bank classification); 41.7% jobless; 31.1% living in couple with both partners working | The main barrier to ART initiation was not disclosing HIV status to a partner. Non-disclosure was associated with sub-Saharan African origin, being single, being diagnosed with HIV late in pregnancy, booking appointments late, and having a partner with an unknown or HIV-negative status. |
| 15 | Jerome (2011) | Brazil | QL | 18 | Standard ART | Adult prevalence rate 0.3% (UNAIDS 2012) | Brazil is upper-middle income according to World Bank; this cohort is relatively low SES for Brazil; all living below poverty line; only 17% finished high school; 39% ever formally employed | Factors enabling use of HIV services included positive perceptions of medical institutions (e.g., health worker attitudes and welcoming of children accompanying women). Fear of stigma was the main reason for non-disclosure. |
| 16 | Kanjipite (2012) | Zambia | QT | 2,759 | PMTCT prophylaxis (regimen not stated) | Prevalence among women16.1% and adult prevalence rate14.2% (DHS 2007) | Unclear (source is an abstract with little detail on characteristics of the cohort); Zambia is classified as lower-middle income by World Bank | The main enabler of ART initiation was couple counseling and testing. |
| 17 | Kasenga (2010) | Malawi | MM | Not stated | sdNVP | 14% among women (in article) | Low (among the poorest districts in Malawi); Malawi classified as low-income according to World Bank | The main barrier to accessing HIV services was negative health worker attitudes, which caused some women to prefer home delivery with a traditional birth attendant. |
| 18 | Kim (2012) | Malawi | QT | 1,688 | Option A | Adult prevalence rate 12% (in article) | Unclear - SES characteristics not captured; Malawi is a low-income country according to World Bank | Enablers of ART adherence were community health worker involvement and a partner not being involved in the woman’s treatment. |
| 19 | Kirsten (2011) | Tanzania | QT | 122 | Option A | Overall prevalence 6%; among pregnant women estimated 10-16%; overall prevalence at site 8%, and among pregnant women at site 18% (in article) | Tanzania is classified as low-income country by World Bank; this cohort - median 7 years of education; majority reported no income-generating activity | Refusal to initiate ART was associated with age (≤23 years old), gestational age (≤24.5 weeks at enrollment), and not having an income-generating activity. Most barriers to ART adherence related to health services (e.g., incorrectly indicated dates for the next visit, neglected dispensation of ARVs, or staff interrupting prophylaxis). |
| 20 | Kohler (2012) | Kenya | QT | 615 | PMTCT prophylaxis (regimen not stated) | National adult prevalence rate is 6.4%; 8% for women (DHS 2008/09) | Unclear, study is an abstract and provided little detail; Kenya is a low-income country according to World Bank | Enablers of initiation included higher education level, PMTCT knowledge, and partner HIV testing. |
| 21 | Kreitchmann (2012) | Latin America | QT | 393 | Option B  Standard ART | Multi-country study: Argentina, Brazil, Peru | Overall median 8.3 years of education, 63.9% not gainfully employed outside the home; Argentina, Brazil, and Peru are upper-middle income by World Bank | Barriers to pre- and postpartum adherence included forgetting (which was more common postpartum), being away from home, changes in daily routines, and running out of medication. Tobacco use was also associated with non-adherence. |
| 22 | Kuonza (2010) | Zimbabwe | QT | 212 | sdNVP | Adult prevalence 15.6%, 4th highest in world (in article) | 70.8% had achieved at secondary education; 29.7% gainfully employed; Zimbabwe is classified as low-income by World Bank | The main enablers of adherence were disclosure to a partner and health facility delivery. Barriers to adherence were forgetting to take medications, misplacing medications, and being advised against it by one's church. |
| 23 | McDonald (2011) | Australia | QL | 16 | Option A | Low, women make up 8.4% of the pop of PLHIV (in article); 0.2% (UNAIDS 2011) | Australia is a high-income country (World Bank); cohort of 16: 5 - tertiary education, 8 completed secondary, 3 completed Year 9 or above | Barriers to treatment included a perceived impersonality of health workers' approach (e.g., treating the disease, not the person); concern about the impact of ART on the child; and psychological distress when taking ARVs (e.g., as a reminder of HIV status and related stigma). |
| 24 | Mellins (2008) | U.S. | QT | 309+ | PMTCT prophylaxis (regimen not stated)  Standard ART | National prevalence rate for gen pop is 0.6% (2009); authors note that HIV prevalence in women in the US is highest among disenfranchised, ethnic minority, empoverished women | Study in US (high income country by World Bank classification); majority of cohort had at least a 10th grade education and apprx half lived on incomes less thatn $10k USD per year | The main enabler of prepartum adherence was the woman's desire to protect her unborn child; lower postpartum adherence may have related to this issue being less of a concern after delivery. Non-adherence was associated with alcohol use. |
| 25 | Mepham (2011) | South Africa | MM | 199 | Option B | 18.9% adult prevalence (UNAIDS 2009); 30% prevalence among pregnant women in South Africa (Dean article) | South Africa is classified as upper-middle income by World Bank; women in the study were all Zulu and attended the KwaMsane clinic in Umkhanyakude District, KwaZulu-Natal | Barriers to prepartum adherence included misunderstanding (e.g., of dosage or instructions); a disrupted personal routine; one’s cell phone being turned off; forgetting to take medications; concern about possible domestic violence; and lack of food or water. Postpartum barriers included relatives taking pills. |
| 26 | Muchedzi (2010) | Zimbabwe | MM | 147 | sdNVP; onward referral to HIV clinic | Among highest infection rates in the world; Prevalence among pregnant women attending ANC in 2007 was 15.6% (in article) | 83% had attained secondary education, 58% were self-employed; one third of participants reported competing life priorities (earning livelihoods and seeking food and shelter) as barriers to accessing HIV care; Zimbabwe is low-income according to World Bank; | Barriers to non-registration at HIV clinics were long waiting lines, long registration processes, and/or competing life priorities. Long wait times were also barriers to prepartum adherence. Membership in an HIV support group was associated with understanding of the referral process and participation in HIV care and treatment. |
| 27 | Myer (2012) | South Africa | QL | 221 | Standard ART | Local antenatal seroprevalence was 10% (in article); | 64% unemployed; median years of schooling 8-10%; 27% at medium to high risk of psychosocial distress; South Africa classified as upper-middle income by World Bank | Experience of counseling and a woman's desire to protect her unborn child were both enablers of ART initiation. The main barrier to initiation was reluctance to commit to a lifelong treatment plan, while the main barrier to adherence was non-disclosure to a partner or family member. |
| 28 | Nassali (2009) | Uganda | QL | 289 | Postnatal care (regimen not stated) | Women infected more than men (in article); 16% prevalence among women delivering at the hospital in the study (in article); 6.3% adult prevalence in 2006 (UNAIDS) | 58% of mothers were employed; 52% had phone access; no education data; Uganda is low-income according to World Bank | The main barrier to adherence was non-disclosure. Factors positively associated with adherence postpartum were previous adherence to PMTCT, age (<25 years), access to a phone (for younger mothers), and Christianity (for older mothers). |
| 29 | O'Gorman (2010) | Malawi | QL | 70 | sdNVP | In 2005 overall prevalence 12%, rate in antenatal women was 16.9%, prevalence lovwe in rural than urban or semi-urban areas (in article) | Did not capture any sociodemographic data; Malawi is low-income according to World Bank | Barriers to ART initiation included fear of divorce or stigma after disclosure, ill treatment by health workers, distance to health facilities (in comparison to traditional birth attendants), and traditional birth attendants running out of medication. In addition, grandmothers played a central role in deciding where women would deliver. |
| 30 | Peltzer (2011) | South Africa | QT | 139+607 | Option A | 18.8% adult prevalence in 2011 (UNAIDS) | Only reported on education level of postnatal women (53.6% had Grade 8-11 education) but not for antenatal women; no data on employment; South Africa is classified as upper-middle income by World Bank | Non-adherence to prenatal adherence was associated with being away from home, forgetting to take medications, and sleeping through dose time. The main enablers were disclosure of status without experience of stigma and partner support. |
| 31 | Stinson (2012) | South Africa | QL | 28 | Standard ART | Adult prevalence rate 18.8% in 2007 (UNAIDS) | 82% of HIV+ women were unemployed; did not report on education; women described cost of care as a barrier; South Africa is classified as upper-middle income by World Bank | Barriers to ART initiation included transport costs, fear of job loss (due to time off), wanting more time to disclose to one’s partners before initiation, and wariness of life-long ART and side effects. The main enabler of initiation was a desire to protect one’s child. Postpartum, negative health worker attitudes were a barrier, while a woman's positive attitude towards treatment was an enabler. |
| 32 | Ujiji (2011) | Kenya | QL | 28+9 | Standard ART | Close to 20% prevalence at antenatal clinics in Busia (one of the sites) (in article); national prevalence rate for gen pop is 6.4%; 8% for women (DHS 2008/09) | Low, average daily household income in Busia was $7 and in Kibera less than $2 (in article); Kenya is a low-income country according to World Bank | Barriers to ART adherence included fear of stigma (and having to hide medication), and constant supervision by the woman's family (particularly mother-in-laws who typically accompany daughter-in-laws for all pregnancy-related care and deliveries). Concern about unintended disclosure at health facilities contributed to women missing HIV clinic appointments, running out of ARVs, and delivering babies at home or with a traditional birth attendant. |
| 33 | Varga (2011) | South Africa | QL | 100 | sdNVP  Continuing care | 15.8% of pregnant adolescents in South Africa in 2005 (in article); during study period (2002-2003) Limpopo's antenatal prevalence was 15.6% wheras the national prevalence was 26.5% (in article) | Low- province is mostly rural (79% of pop) and high levels of unemployment (77%); 57% of those who work earn leass than $142USD a month; | Barriers to HIV testing (and, therefore, ART initiation) were associated with negative health care provider experiences and fear of stigma or a breach of confidentiality after disclosure. Positive experiences with lay counselors promoted used of HIV services postpartum. |
| 34 | Watson-Jones (2012) | Tanzania | MM | 403 | PMTCT prophylaxis (regimen not stated)  Standard ART | Prevalence among pregnant women in Mwanza 8.4% in 2008 (in article) | 90.6% had at least primary school education; 47.6% were housewives; 48.6% reported having unskilled work, 3.7% had skilled work; Tanzania is classified as low-income country by World Bank | Barriers to attending an HIV clinic (when referred) included not understanding why it was important, fear of disclosure, distance, wanting to wait until after delivery, and feeling too healthy. Poor attendance (once enrolled) was associated with non-disclosure, fewer than three ANC visits, poor adherence to PMTCT, and Muslim religion (as opposed to Christian). |

1. Key: MM = mixed (qualitative and quantitative) methods; QL = qualitative method(s); QT = quantitative method(s) [↑](#footnote-ref-1)
