# Supplementary material for: A Systematic Review of Individual and Contextual Factors Affecting ART Initiation, Adherence, and Retention for HIV-Infected Pregnant and Postpartum Women
Source: PLoS One. 2014 Nov 5;9(11):e111421. doi: 10.1371/journal.pone.0111421 (PMC4221025; doi:10.1371/journal.pone.0111421)
Supplement: Table S3 — Findings by Level, Theme, and Study. (DOCX) [file pone.0111421.s003.docx]

# Findings by Level, Theme, and Study

| **Level of Influence** | **Thematic Subcategory 1** | **Thematic Subcategory 2** | **Thematic Subcategory 3** | | **Study Identification Number** | | | | | **Number of Studies Reporting a Finding** |
| --- | --- | --- | --- | --- | --- | --- | --- | --- | --- | --- |
|  |  |  |  |  | **Initiation** | | **Adherence** | **Retention** | |  |
| **INDIVIDUAL** | |  |  | |  | |  |  | | 65 |
|  | **Demographic** |  |  | |  | |  |  | | **11** |
|  |  | Age and education |  | |  | |  |  | | 8 |
|  |  |  | Older  [enabler] | |  | | *2* |  | | 1 |
|  |  |  | Younger  [barrier] | | 19 | | 8, 21 |  | | 3 |
|  |  |  | Higher education level [enabler] | | *20* | | *2, 4* |  | | 3 |
|  |  |  | Lower education level [barrier] | |  | | 10 |  | | 1 |
|  |  | Location |  | |  | |  |  | | 3 |
|  |  |  | Rural location | |  | | 2, 32 |  | | 2 |
|  | **Knowledge** |  |  | |  | |  |  | | **7** |
|  |  | Poor |  | |  | |  |  | | 3 |
|  |  |  | PMTCT | | |  |  | 11, 34 | | 2 |
|  |  |  | Medication | |  | | 25 |  | | 1 |
|  |  | Sufficient |  | |  | |  |  | | 4 |
|  |  |  | PMTCT | | *20* | | *31* | *5* | | 3 |
|  |  |  | Referral process | |  | |  | *26* | | 1 |
|  | **Fears or Aspirations** | |  | |  | |  |  | | **17** |
|  |  | Fears |  | |  | |  |  | | 8 |
|  |  |  | Job loss | | 31 | |  |  | | 1 |
|  |  |  | Denial of HIV | | 6, 10, 31 | | 23 | 34 | | 5 |
|  |  |  | Negative impact of ART on child | |  | | 23, 24 |  | | 2 |
|  |  | Aspirations |  | |  | |  |  | | 9 |
|  |  |  | Desire to remain healthy | |  | | *12* |  | | 1 |
|  |  |  | Desire to protect child | | *27, 31* | | *12, 24* |  | | 4 |
|  |  |  | Reluctance to commit to lifelong treatment | | 27, 31 | |  |  | | 2 |
|  |  |  | Perceived conflict between role as homemaker and needs as patient | |  | | 1, 32 |  | | 2 |
|  | **Practical Demands** |  |  | |  | |  |  | | **11** |
|  |  | Too ill |  | |  | |  | 34 | | 1 |
|  |  | Holiday or away from home |  | |  | | 25, 30 |  | | 2 |
|  |  | No income generation |  | | 19 | |  |  | | 1 |
|  |  | Cell phone off (no text messages) |  | |  | | 25 |  | | 1 |
|  |  | Access to phone |  | |  | |  | *28* | | 1 |
|  |  | Scheduling problems or competing life priorities | | |  | | 9, 12, 19 | 26 | | 4 |
|  |  | Lack of food/water/income |  | |  | | 1, 25 |  | | 1 |
|  | **Beliefs** |  |  | |  | |  |  | | **8** |
|  |  | Feeling too healthy to go for test/treatment |  | | 2, 31 | |  | 34 | | 3 |
|  |  | Positive outlook (happy) |  | |  | | *3* |  | | 1 |
|  |  | Religion |  | |  | |  |  | | 4 |
|  |  |  | Enabler | |  | |  | *28, 34* | | 2 |
|  |  |  | Barrier | |  | | 22 | 5 | | 2 |
|  | **Behavior** |  |  | |  | |  |  | | **12** |
|  |  | Drugs/alcohol |  | |  | | 4, 8, 21 |  | | 5 |
|  |  | Misplacing medication |  | |  | | 22 |  | | 1 |
|  |  | Forgetting medication |  | | 10 | | 12, 19, 21, 27 |  | | 6 |
|  |  |  |  | |  | |  |  | |  |
| **INTERPERSONAL** | |  |  | |  | |  |  | | 23 |
|  | **Spouse/Partner** |  |  | |  | |  |  | | **19** |
|  |  | Dependence or permission needed |  | | 10, 29 | |  | 11 | | 4 |
|  |  | Domestic violence |  | |  | | 25 | 11 | | 2 |
|  |  | Disclosure to partner |  | |  | |  |  | | 8 |
|  |  |  | Non-disclosure | | 14, 31 | |  | 11, 13, 34 | | 6 |
|  |  |  | Disclosure | |  | | *19* | *11* | | 2 |
|  |  | Partner involvement |  | |  | |  |  | | 5 |
|  |  |  | Partner not involved | |  | |  | 6, *18* | | 2 |
|  |  |  | Partner involved | | *16, 20* | | *30* |  | | 3 |
|  | **Family** |  |  | |  | |  |  | | **4** |
|  |  | Family support |  | |  | |  |  | | 3 |
|  |  |  | Negative support | | 29 | | 32 |  | | 2 |
|  |  |  | Positive support | |  | | *1* |  | | 1 |
|  |  | Relatives stealing ART pills |  | |  | | 25 |  | | 1 |
|  |  |  |  | |  | |  |  | |  |
| **COMMUNITY** | |  |  | |  | |  |  | | 16 |
|  | **Stigma and Disclosure** | |  | |  | |  | **16** | | **45** |
|  |  | Stigma |  | | 29, 33 | | 8, 12, 25, 27, 32 | 6, 7, 11, 28, 34 | | 13 |
|  |  | Disclosure |  | |  | |  |  | | 3 |
|  |  |  | Disclosure without stigma | |  | | ***22, 30*** |  | | 2 |
|  |  |  | Non-disclosure | |  | | 1 |  | | 1 |
|  |  |  |  | |  | |  |  | |  |
| **STRUCTURAL** | |  |  | |  | |  |  | | 42 |
|  | **Use of Health Services** | |  | |  | |  |  | | **9** |
|  |  | First pregnancy registration |  | |  | |  | ***13*** | | 1 |
|  |  | Disengagement late in pregnancy (<30 days) | | |  | |  | 2 | | 1 |
|  |  | Late or low attendance at ANC |  | |  | |  |  | | 3 |
|  |  |  | Low attendance | |  | |  | 13, 34 | | 2 |
|  |  |  | Late (third trimester) | |  | |  | ***18*** | | 1 |
|  |  | New HIV infection diagnosis |  | |  | | ***18*** |  | | 1 |
|  |  | Previous prenatal care |  | |  | |  | ***28*** | | 1 |
|  |  | Delivery in health facility |  | | ***10, 20*** | |  |  | | 2 |
|  | **Health Care Workers** | |  | |  | |  |  | | **18** |
|  |  | Negative attitudes |  | | 17, 33 | | 23, 29, 31 | 11, 13, | | 7 |
|  |  | Positive attitudes |  | | **15** | |  |  | | 1 |
|  |  | Status of confidentiality |  | |  | |  |  | | 3 |
|  |  |  | Actual or possible breach | |  | | 1 | 7 | | 3 |
|  |  | Treatment support or counseling |  | | *27* | | *12* | *3* | | 3 |
|  |  | Support group or social support |  | | *19* | |  | *26* | | 2 |
|  |  | Community health worker involvement | | |  | |  | *18* | | 1 |
|  |  | Role of traditional birth attendant |  | | *29* | |  |  | | 1 |
|  | **Access to Health Services** | |  | |  | |  |  | | **12** |
|  |  | Long queues |  | 19 | | | 1, 26 |  | 5 | |
|  |  | Transport problems (including cost) | | 29, 31, 34 | | | 19 | 11 | 6 | |
|  |  | Lack of free treatment |  |  | | | 12 |  | 1 | |
|  | **Treatment** |  |  |  | | |  |  | **5** | |
|  |  | Non-provision or problems receiving sdNVP and ART at health facility |  |  | | | 19 |  | 1 | |
|  |  | Receiving other treatment (e.g., for tuberculosis) | |  | | | *2* |  | 1 | |
|  |  | Pre-delivery prophylaxis |  |  | | | *19* | *13* | 2 | |
|  |  | Successful PMTCT pre-natal |  |  | | |  | *28* | 1 | |

For Study Identification Numbers: Italicized text = enabler; Plain text = barrier
