# Supplementary material for: A Systematic Review of Individual and Contextual Factors Affecting ART Initiation, Adherence, and Retention for HIV-Infected Pregnant and Postpartum Women
Source: PLoS One. 2014 Nov 5;9(11):e111421. doi: 10.1371/journal.pone.0111421 (PMC4221025; doi:10.1371/journal.pone.0111421)
Supplement: Table S4 — Outcome Measures Used in the Studies. (DOCX) [file pone.0111421.s004.docx]

# Outcome Measures within the Reviewed Studies

| **No.** | **First Author** | **Main Outcome** | **Outcome Measure** |
| --- | --- | --- | --- |
| 1 | Awiti-Ujiji | Adherence | Self-report – women described challenges in adhering to treatment after giving birth. No measure of “adherence” or “non-adherence”, just reflection on factors that make postpartum adherence challenging. |
| 2 | Ayuo | Both | There were two: (1) Self-reported adherence – perfect adherence was having taken all pills in last seven days. (2) Disengagement - Early disengagement was defined as no contact for 30 consecutive days during pregnancy, with return prior to delivery. Late disengagement was defined as no contact within 30 days prior to delivery. Authors say that findings cannot be directly compared with retention data from the program or others because of their conservative definition of disengagement and the short follow-up period required for PMTCT. |
| 3 | Aziz | Retention | Abstract in Sexually Transmitted Infections; measured uptake of VCT, returning to collect results, starting AZT, completion of regimen, and dropout. AZT regimen is 300 mg twice daily from 36 weeks gestation until labor, one tablet at onset of labor, and then every 3 h [during?] delivery. Measure of adherence to AZT was “consent”, “completion”, “currently receiving”, or “dropped out”. |
| 4 | Bardeguez | Adherence | Self-report – perfect adherence defined as patient attended clinic visit, completed adherence form, and taken all ARVs over last four days. Imperfect adherence if one or more dose missed or incomplete information on any day in the form. Adherence outcome considered missing if patient missed clinic appointment or did not submit form. Second measure was when the subject last missed a dose (within last week). |
| 5 | Boateng | Retention | Medical records – default defined as consistently missing two or more PMTCT appointments in the previous two months Authors describe this as adherence to ART |
| 6 | Bwirire | Retention | Describes retention in PMTCT program and reasons for LTFU based on observation and FGDs. Identifies nine themes/reasons for LTFU. |
| 7 | Chinkonde | Retention | Self-report during in-depth interviews; describes reasons for dropping out of follow up visits that are part of a PMTCT program. The program follows women and their infants monthly from 6 weeks after delivery until the infant is 18 months old. Cohort included women who had accepted to attend follow-up visits and had been diagnosed in the last 6 months. Measure of retention: if a woman failed to attend a scheduled program visit, she was classified as a “drop-out”. |
| 8 | Cohn | Adherence | Self-reported adherence was defined as not missing any doses for four days prior (recent adherence) and not missing any doses for three or more months (extended adherence). |
| 9 | Dean | Adherence | Measured patterns of use and dialogue of an SMS supportive intervention among seven recently diagnosed HIV+ pregnant women. The intervention aimed to promote adherence to ART. Article did not report on actual adherence levels. During post-intervention interviews, some women described the intervention as having influenced her decision to start ARVs or supported with difficult decisions. |
| 10 | Delvaux | Adherence (sdNVP) | Adherence data was collected from PMTCT clinic patient registers and confirmed through self-reported. Adherence was defined as mother-infant pairs ingesting sdNVP at the recommended time. Non-adherence was defined as mother-infant pairs not ingesting sdNVP, or doing so at the wrong time. |
| 11 | Duff | Retention | Self-report through in-depth interviews and FGDs. Retention/adherence measured as (1) eligible but never enrolled for HAART; (2) enrolled but never began HAART; (3) Defaulted HAART; (4) Taking HAART (without reported interruption) Women described barriers to accessing HAART. |
| 12 | Ekama | Adherence | Adherence data was collected through self-report and measured as number of doses taken as a percentage of the prescribed dosages. |
| 13 | Ferguson | Retention in care | Routine hospital data – client attrition determined by assessment of registration at HIV clinic within six months of HIV diagnosis during pregnancy-related services. |
| 14 | Jasserson | Adherence | Based on review of review of clinician/midwife competed routine questionnaires. Measure of use of ART defined as “non-optimal”: no ART during pregnancy, and/or late initiation of ART >31 weeks gestational, and/or detectable viral load near delivery > or = 50 cp/ml; vaginal delivery despite viral load above 400 cp/ml; no intrapartum prophylaxis; no neotnatal prophylaxis; and breastfeeding. Authors also considered severe immunosuppression near delivery (CD4 count < 200). Combined indicator for antenatal PMTCT was coded non-optimal if one or more was reported: no treatment during pregnancy, late initiation of ART, or viral load greater than 1,000 copies/ml. |
| 15 | Jerome | Initiation | Self-report through interviews; women described experiences with treatment and care, and factors affecting acceptance and use of treatment. Adherence or retention not measured. |
| 16 | Kanjipite | Initiation | Abstract for AIDS2012; measured increase in uptake of ART by pregnant woman who attended VCT with their partner compared to those who did not come with a partner during previous years. |
| 17 | Kasenga | Initiation | ANC, PMTCT and delivery registers, self-report through structured questionnaires and FGDs; describes use of maternity care and factors that affect it. Does not provide quantitative measures. |
| 18 | Kim | Retention | Describes a pilot intervention using CHW to support completion of PMTCT cascade. It measured retention, utilization of services, and outcomes and compares it to available data for pre-intervention period at same sites. Information was taken from patient mastercards (completed by CHW) and patient registers. Measures of retention/use of services were: Accessed antenatal care; Test for HIV Infection and Enroll in Care; Determined ART Eligibility (already taking or not; needed CD4 test, received CD4 test results); Initiate ART/PMTCT Prophylaxis (nevirapine only, nevirapine and AZT only, full combination prophylaxis, ART for mothers health, none, unknown); Follow up (Place of delivery); Access Postnatal Care (Infant received prophylaxis by type; infant feeding choice after birth); Infant Test for HIV Infection and Enroll into Care (Infant received PCR test and CPT); Determine HIV Status of infant (test result returned from lab and outcome); Initiate ART (enrolled in clinic, started on ART, age at initiation); Follow up (Mother-infant part still being followed in program). |
| 19 | Kirsten | Adherence | Adherence measures assessed: (1) drug collection (during pregnancy) – possession ratio generated from # of AZT doses collected divided by targeted number of doses; (2) sdNVP (during delivery) – reported by nurse or mother; (3) intra and postpartum AZT and 3TC and AZT for newborns (during delivery and after delivery before discharge) – dividing hours covered by dispensed drugs by total hours of hospitalization until delivery; (4) dispensation by staff (during/after delivery) – absolute numbers of women and infants supplied with correct amount of drugs to take home |
| 20 | Kohler | Adherence | Community-based surveys with women who delivered in prior year – uptake of ANC and maternal/infant AR; resource is an abstract and does not offer detailed definitions for measures. Provides data for those who “took ART for PMTCT” as percentages antenatally, at labor, postpartum, or all three times. Measures receipt of ARVs during labor for infants. |
| 21 | Kreitchmann | Adherence | Self report – Percent adherence derived by number of doses missed divided by total number of expected doses. Short-term adherence was measured as number of missed doses in the three days before the study visit. Long-term adherence was measured by the last time an ARV dose was missed, with the response options: never, during the previous two weeks, during the last month, over a month ago, or do not remember. |
| 22 | Kuonza | Adherence | PMTCT patient registers - adherence was defined as mother-infant pairs ingesting sdNVP at the recommended time. Non-adherence was defined as mother-infant pairs not ingesting sdNVP, or doing so at the wrong time. |
| 23 | McDonald | Adherence | Self-report (interviews) – discussed beliefs and reasons for taking/not taking ART during pregnancy or giving it to their baby. |
| 24 | Mellins | Adherence | Self-report (adherence questionnaires) – Short-term adherence was measured by number of missed doses in the two days before the study visit. Also reported on the last time an ARV dose was missed, with the response options: during the past week, during the past two weeks, during the past month, during the past three months, more than three months ago, or never.Complete adherence defined as composite of total adherence to all ART meds taken in past two days and no missed doses within the past month. |
| 25 | Mepham | Adherence | Pill counting at 28^th^ week, 29^th^ week, and thereafter four weekly until one-week postpartum (for short course patients) or four weeks after breastfeeding cessation around six months postpartum. Pharmacists asked for reasons for poor adherence. Good adherence defined as over 95%, calculated by total number of pills taken by participant divided by total number of pills expected to be taken (for entire prophylaxis period). |
| 26 | Muchedzi | Retention in care | Interviews, FGDs, and review of ANC registration book; measure was registration at an HIV clinic following referral from the PMTCT program. |
| 27 | Myer | Initiation | Describes pilot program for rapid initiation of ART. Data take from patients records and in-depth interviews. Measure was initiating ART during pregnancy. Did not measure adherence after initiation. |
| 28 | Nassali | Retention in care | Adherence to a postnatal PMTCT program was measured as the proportion of mothers who honored scheduled appointments by their eighth week postpartum. |
| 29 | O’Gorman | Did not provide adherence data, just factors that may affect | Semi-structured interviews and FGDs – qualitative gathering of views about swallowing nevirapine or bringing the baby for nevirapine |
| 30 | Peltzer | Adherence | Self-report (structured questionnaires) – complete adherence measured as adherence to appropriate medication schedule in the four days preceding the interview or prior to delivery. Reported on dose the morning of the interview, 2 days prior, 4 days prior, and never missed a dose during this pregnancy prior to interview. |
| 31 | Stinson | Utilization of services (use or non-use of ART) | Self-report (structured interview) – identified barriers to swift initiation of ART in pregnancy and postpartum use of ART |
| 32 | Ujiji | Adherence | Interviews, unstructured dialogue. Findings describe the experience of HIV+ women on ART who choose to become pregnant. Women described being non-adherent to PMTCT as missing appointments, delayed or missed taking HIV medicines, and planning to having home deliveries. |
| 33 | Varga | Initiation | Key informant narrative workshops; FGDs, community based surveys. Describes factors affecting use of services. |
| 34 | Watson-Jones | Retention | Data collected from health worker interviews, observation of PMTCT services, and cohort study of HIV+ women recruited at maternity wards (antenatal card and interviews). Measures included: location/timing of testing and diagnosis (PMTCT services during pregnancy, at maternity ward before delivery, at maternity ward after delivery); received referral to HIV clinic; attended HIV clinic prior to delivery; had evidence of CD4 test result; were seen at the four month follow-up visit; started HAART within four months of delivery. Describes factors affecting retention in service and attendance at different points along the cascade. |
